# Supplementary material for: CXCL5 activates CXCR2 in nociceptive sensory neurons to drive joint pain and inflammation in experimental gouty arthritis
Source: Nat Commun. 2024 Apr 16;15:3263. doi: 10.1038/s41467-024-47640-7 (PMC11021482; doi:10.1038/s41467-024-47640-7)
Supplement: Supplementary file 1 — Supplementary Information [file 41467_2024_47640_MOESM1_ESM.pdf]

Supplementary Materials for

CXCL5 activates CXCR2 in nociceptive sensory neurons to drive joint pain and inflammation in experimental gouty arthritis

Chengyu Yin et al.

Corresponding authors: Boyi Liu boyi.liu@foxmail.com; Chuan Wang wangchuan@hebmu.edu.cn; Jianqiao Fang fangjianqiao7532@163.com; Hailong An hailong_an@hebut.edu.cn.

This file includes:

Fig. S1 to S11

Tables S1-S3

Supplementary figures

Suppl Fig. 1

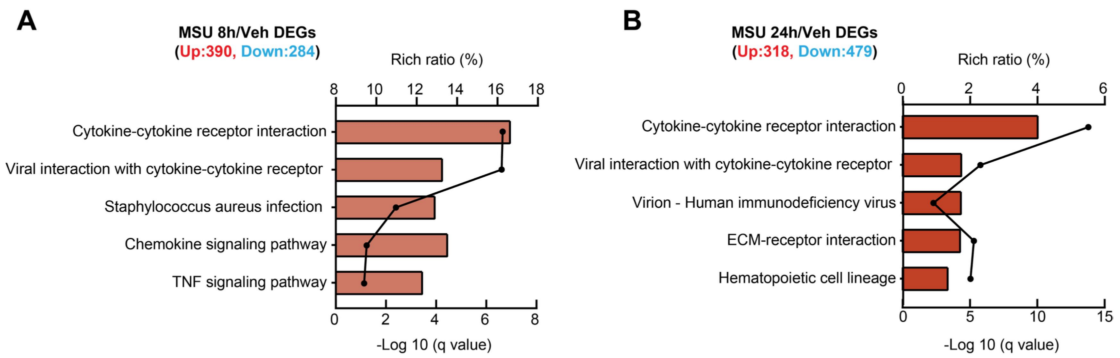

Suppl. Fig. 1 DEGs analyzed by KEGG in ankle joint of gout model mice. (A) Top five enriched signaling pathways at 8 h time point. (B) Top five enriched signaling pathways at 24 h time point.

Suppl Fig. 2

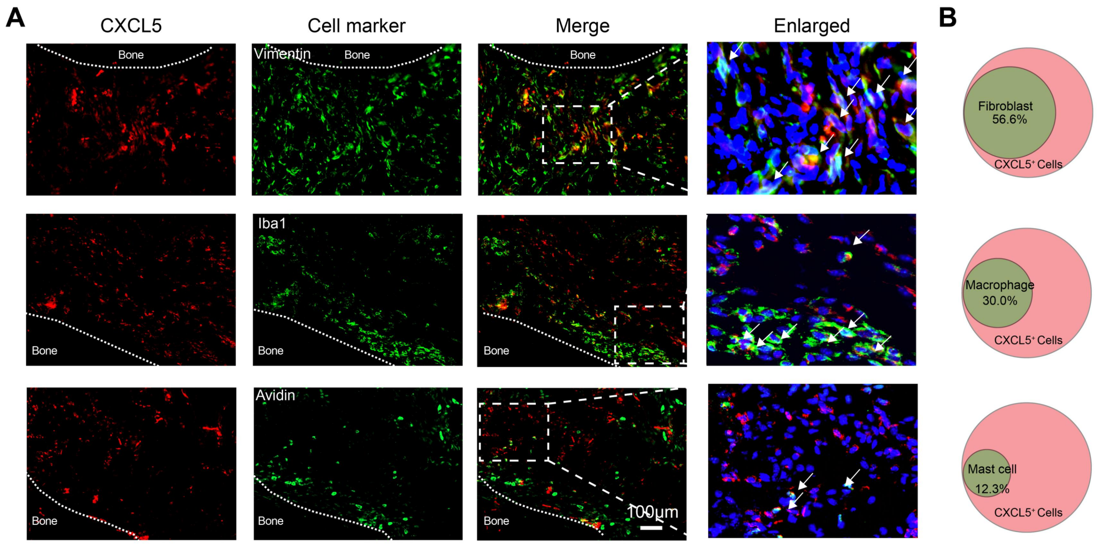

Suppl. Fig. 2 Cell sources for CXCL5 production in the inflamed ankle joint of gout model mice. (A) Immunostaining of periarticular tissues showing CXCL5 co-labeling with markers for fibroblasts (vimentin), macrophages (Iba1) and mast cells (avidin) in gout model mice. Scale bar = 100 μm. (B) Venn diagram showing the overlay of fibroblasts, macrophages and mast cells with CXCR5 positively stained ($CXCR5^{+}$) cells.

Suppl Fig. 3

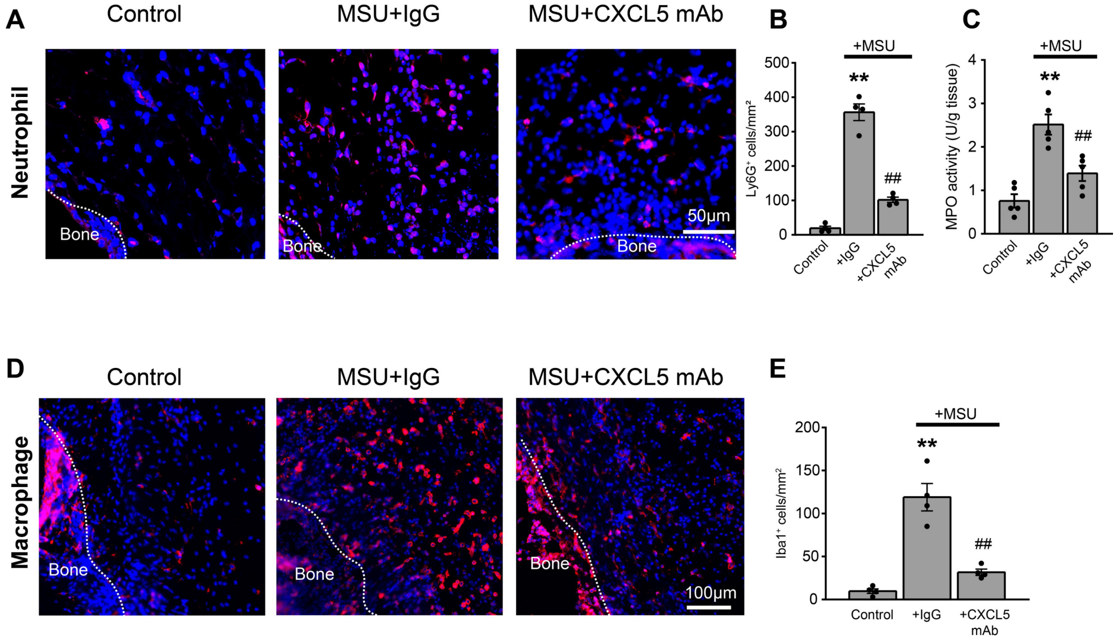

Suppl. Fig. 3 Neutralizing CXCL5 reduced inflammatory cell infiltration in ankle joint of gout model mice. (A) Representative immunostaining pictures showing neutrophils (stained with Ly6G antibody, in red) in periarticular tissues of control and gout model mice. Purple: DAPI staining. CXCL5 or control IgG was intra-articularly injected (3 μg/site) along with MSU or PBS (control group). Scale bar = 50 μm. (B) Summarized number of Ly6G$^+$ cells/mm^2. (C) MPO activity assays of ankle joints from 3 groups of mice. (D) Representative immunostaining pictures showing macrophages (stained with Iba1 antibody, in red) in periarticular tissues of control and gout model mice. Scale bar = 50 μm. (E) Summarized number of Iba1$^+$ cells/mm^2. $^{**}p<0.01$ vs. control. $^{\#\#}p<0.01$ vs. MSU+IgG. One-way ANOVA with Bonferroni's post hoc test was used for statistics. The data are shown as mean ± SEM. The n number, exact p value and statistical results are provided as a Source Data file.

Fig. S4

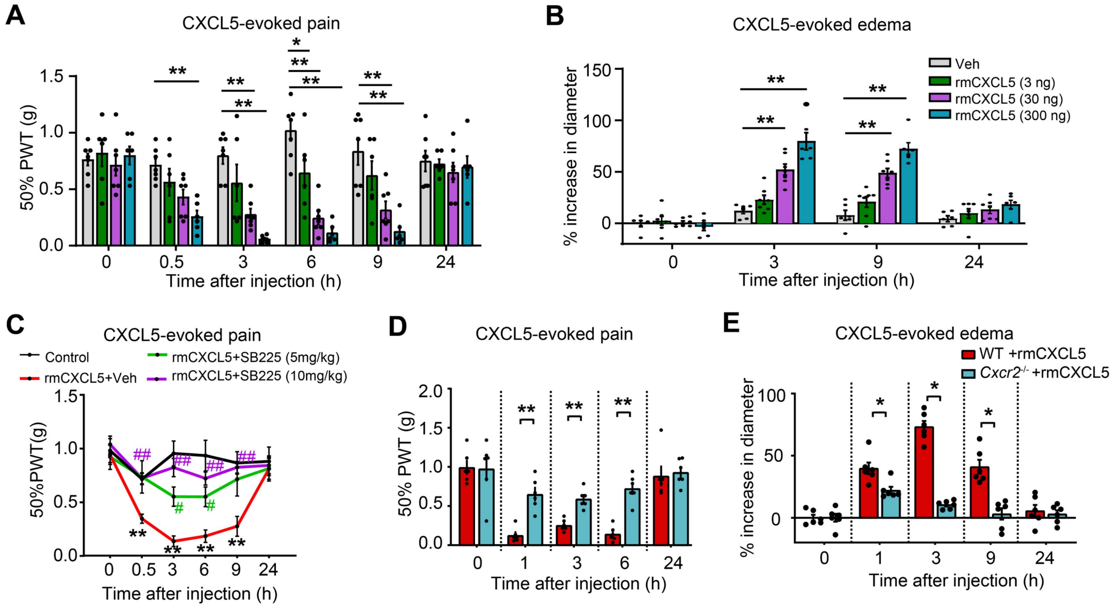

Suppl. Fig. 4 CXCR2 mediates CXCL5–induced mechanical allodynia and joint inflammation in mice. (A) 50% PWT changes upon vehicle (0.1 % BSA in PBS) or different doses of recombinant mouse CXCL5 (rmCXCL5) injection (3, 30 and 300 ng/20 µl) into the ankle joint of naïve mice. (B) % increase in ankle diameter upon vehicle or different doses of CXCL5 injection. (C) Effect of pharmacological blocking CXCR2 with the specific antagonist SB225002 (5 mg/kg) on CXCL5-induced mechanical allodynia. (D) Comparison of CXCL5-induced mechanical allodynia in WT with *Cxcr2*$^{-/-}$ mice. (E) Comparison of CXCL5-induced joint enema in WT with *Cxcr2*$^{-/-}$ mice. **p*<0.05, ***p*<0.01. Two-way ANOVA (repeated measures) with Bonferroni's post hoc test was used for statistics. The data are shown as mean ± SEM. The n number, exact *p* value and statistical results are provided as a Source Data file.

Fig. S5

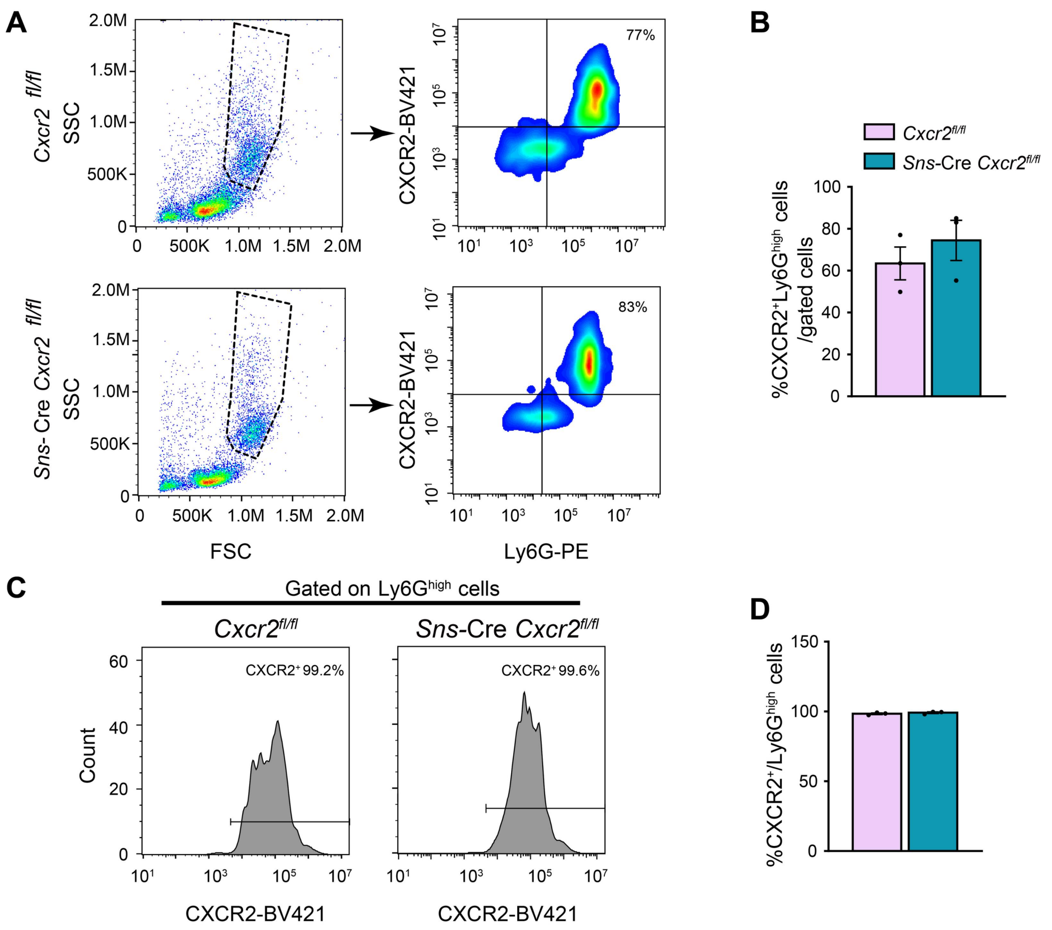

Suppl. Fig. 5 CXCR2 expression is not altered in neutrophils from SNS-Cre Cxcr2$^{fl/fl}$ mice. (A) Left panels indicate the viable monocytes gated through forward scatter (FSC)/side scatter (SSC) plot from the blood of *Cxcr2*$^{fl/fl}$ or SNS-Cre *Cxcr2*$^{fl/fl}$ mice. Right panels indicate the representative flow cytometry plots of Ly6G^{+} and CXCR2^{+} stained cells from the gated cells. (B) Summary of the % of CXCR2^{+}Ly6G^{+} neutrophils among total gated cells from *Cxcr2*$^{fl/fl}$ or SNS-Cre *Cxcr2*$^{fl/fl}$ mice. (C) Histogram data from FACS enrichment of Ly6G^{+} cells. The bar in each graph represents the sorting gate defined as CXCR2 positive event. The percentage of cells counted as positive event is indicated above the bar. (D) Summary of the % of CXCR2^{+} cells among Ly6G^{+} cells as indicated in panel C. Student's unpaired *t* test (two-tailed) was used for analysis in panel B&D. The data are shown as mean ± SEM. The n number, exact *p* value and statistical results are provided as a Source Data file.

Fig. S6

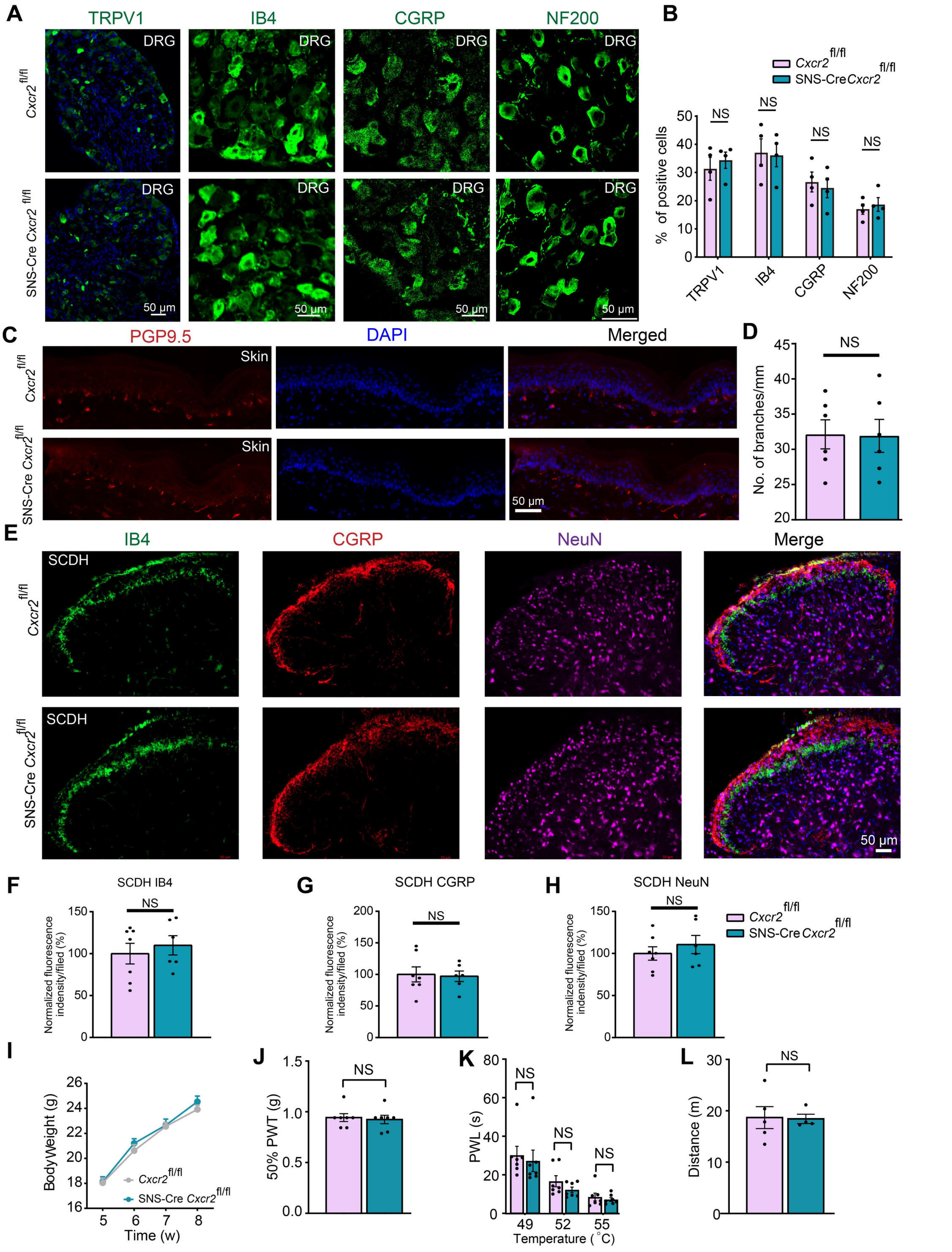

Suppl. Fig. 6 SNS-Cre *Cxcr2*$^{fl/fl}$ mice showed normal percentage of different neuronal populations in DRG and normal peripheral and central innervations. (A) Immunostaining showing the expression of TRPV1, IB4, CGRP and NF200 in DRG neurons from *Cxcr2*$^{fl/fl}$ and SNS-Cre *Cxcr2*$^{fl/fl}$ mice. Scale bars = 50 μm. (B) Comparison of the percentages of positively stained cells between two groups of mice.

(C) Immunostaining of PGP9.5 in glabrous skin from the hind paw. (D) Comparison of the No. of PGP9.5-stained peripheral nerve branches between two groups of mice. Scale bar = 50 µm. (E) Immunostaining of IB4, CGRP and NeuN in spinal cord dorsal horn. (F-H) Comparison of IB4, CGRP and NeuN in spinal cord dorsal horn between two groups of mice. Scale bar = 50 µm. (I-L) Comparisons of body weight (I), 50% PWT (J), PWL (K) and locomotor activity (L) between *Cxcr2*$^{fl/fl}$ and SNS-Cre *Cxcr2*$^{fl/fl}$ mice. Two-way ANOVA with Bonferroni's post hoc test was used for statistics in panel I. Student's unpaired *t* test (two-tailed) was used for others. The data are shown as mean ± SEM. The n number, exact *p* value and statistical results are provided as a Source Data file.

Fig. S7

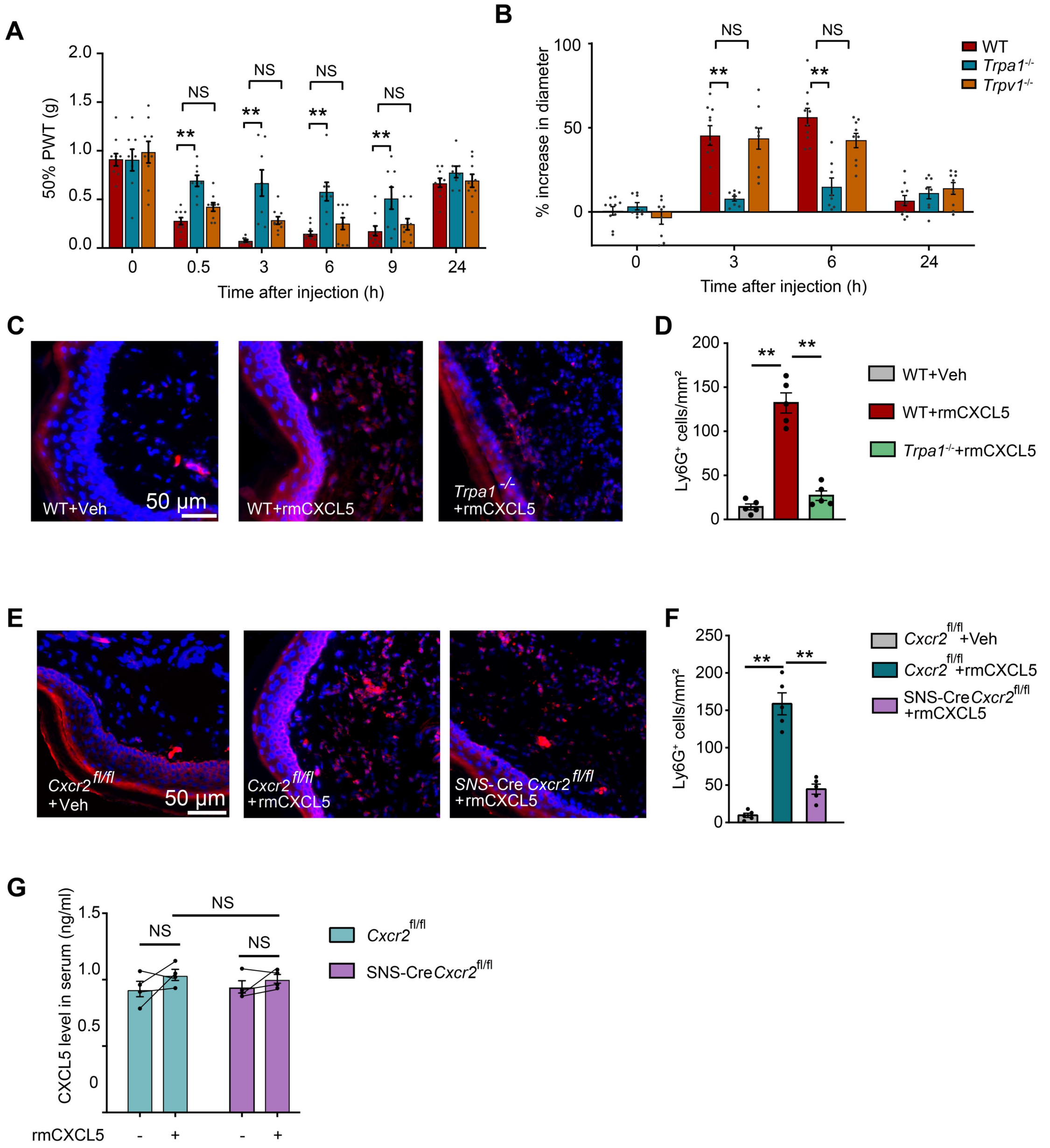

Suppl. Fig. 7 CXCL5 induced mechanical allodynia and joint inflammation is reduced in *Trpa1*$^{-/-}$ *or* SNS-Cre *Cxcr2*$^{fl/fl}$ mice. (A) 50% PWTs measured in WT, *Trpa1*$^{-/-}$ and *Trpv1*$^{-/-}$ mice before and after intraarticular CXCL5 injection (300 ng/site). (B) Ankle joint swelling of WT, *Trpa1*$^{-/-}$ and *Trpv1*$^{-/-}$ mice after intraarticular CXCL5 injection. (C) Representative immunostaining pictures showing neutrophils in hindpaw tissues of WT and T*rpa1*$^{-/-}$ mice 3 h after vehicle or rmCXCL5 injection. Red: Ly6G staining. Purple: DAPI staining. Scale bar = 50 µm. (D) Summarized numbers of Ly6G^{+} cells/mm^{2} as in panel C. (E) Representative immunostaining

pictures showing neutrophils in hindpaw tissues of *Cxcr2*$^{fl/fl}$ and SNS-Cre *Cxcr2*$^{fl/fl}$ mice 3 h after vehicle or rmCXCL5 injection. Scale bar = 50 µm. (F) Summarized numbers of Ly6G^{+} cells/mm^2 as in panel E. (G) ELISA showing serum levels of CXCL5 in *Cxcr2*$^{fl/fl}$ and SNS-Cre *Cxcr2*$^{fl/fl}$ mice before and after CXCL5 (300 ng/site) injection into hindpaws. Serum was collected before and 3 h after CXCL5 injection. $^{**}p$ <0.01. Two-way ANOVA (repeated measures) with Bonferroni's post hoc test in panels A&B. One-way ANOVA with Bonferroni's post hoc test in panels D, F&G. The data are shown as mean ± SEM. The n number, exact *p* value and statistical results are provided as a Source Data file.

Fig. S8

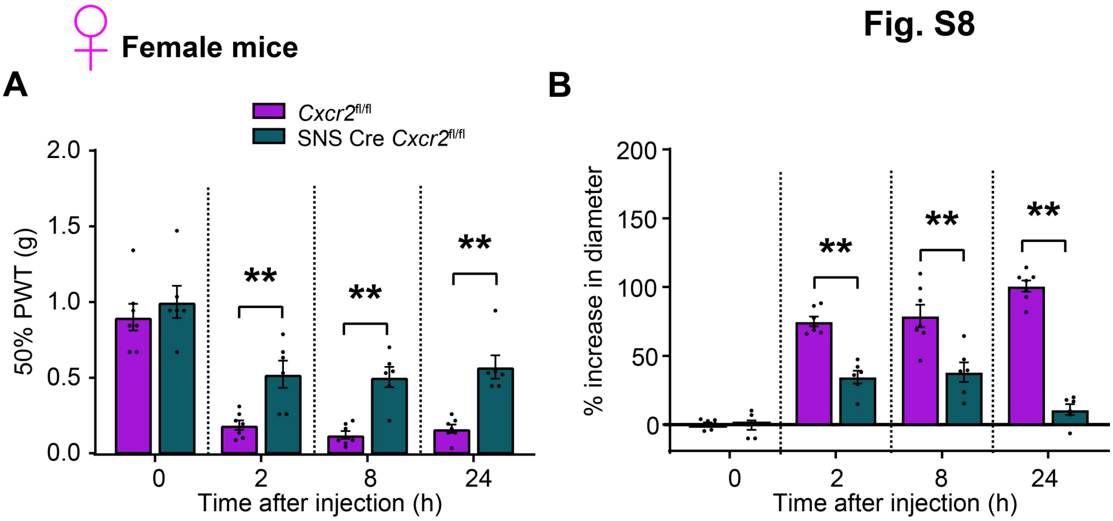

Suppl. Fig. 8 MSU-induced joint pain and inflammation is also reduced in female SNS-Cre *Cxcr2*$^{fl/fl}$ mice. (A) 50% PWTs measured in female *Cxcr2*$^{fl/fl}$ and SNS-Cre *Cxcr2*$^{fl/fl}$ mice before and after intraarticular MSU injection. (B) Ankle joint swelling of female *Cxcr2*$^{fl/fl}$ and SNS-Cre *Cxcr2*$^{fl/fl}$ mice after intraarticular MSU injection. $^{**}p$<0.01. Two-way ANOVA (repeated measures) with Bonferroni's post hoc test. The data are shown as mean ± SEM. The n number, exact *p* value and statistical results are provided as a Source Data file.

Fig. S9

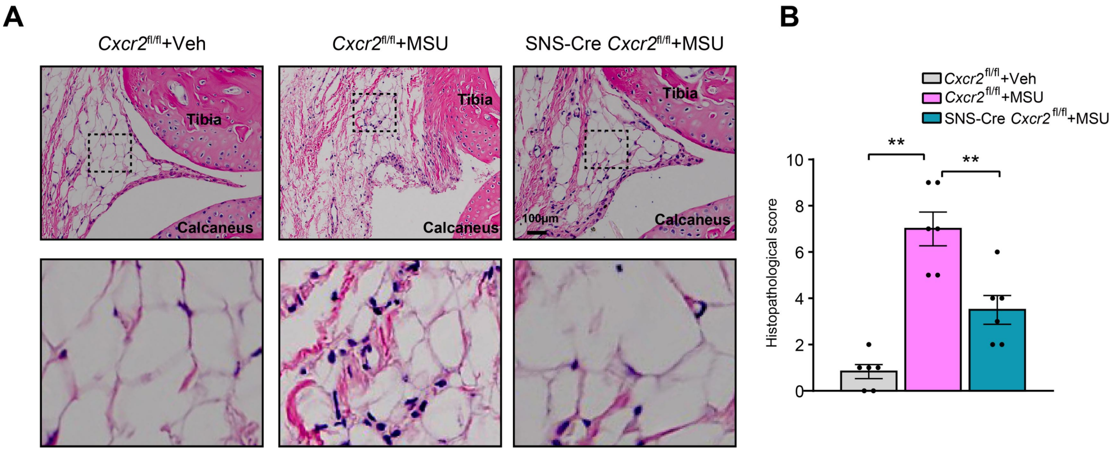

Suppl. Fig. 9 Histopathological examination of ankle joints by H&E staining. (A) Representative H&E staining of ankle joints from *Cxcr2*$^{fl/fl}$ and SNS-Cre *Cxcr2*$^{fl/fl}$ mice treated with vehicle or MSU. The dotted area was further enlarged and shown in panels below. Scale bar = 100 μm. (B) The comparsion of histopathological score of each group. $^{**}p<0.01$. One-way ANOVA with Bonferroni's post hoc test. The data are shown as mean ± SEM. The n number, exact *p* value and statistical results are provided as a Source Data file.

Fig. S10

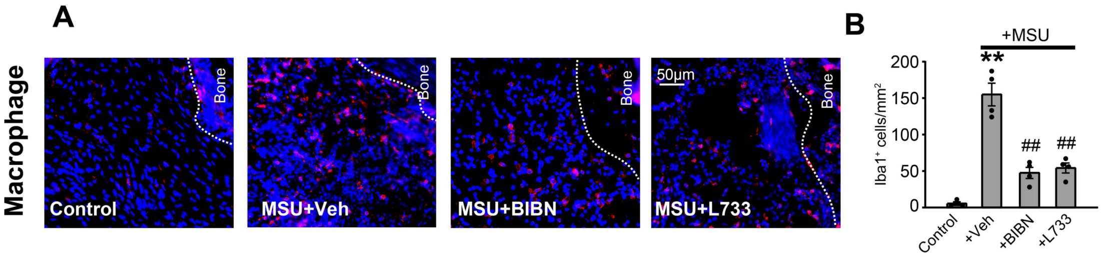

Suppl. Fig. 10 Antagonizing CGRP receptor or SP NK-1 receptor reduced macrophage infiltration in ankle joints of gout model mice. (A) Representative immunostaining pictures showing macrophages in periarticular tissues of control, MSU+veh, MSU+BIBN and MSU+L733060 group of mice. Red: Iba-1 staining. Purple: DAPI staining. Scale bar = 50 μm. (B) Summarized numbers of Iba1^{+} cells/mm^{2} as in panel A. $^{**}p<0.01$ vs. control group. $^{\#\#}p<0.01$ vs. MSU+Veh group. One-way ANOVA with Bonferroni's post hoc test. The data are shown as mean ± SEM. The n number, exact *p* value and statistical results are provided as a Source

Data file.

Fig. S11

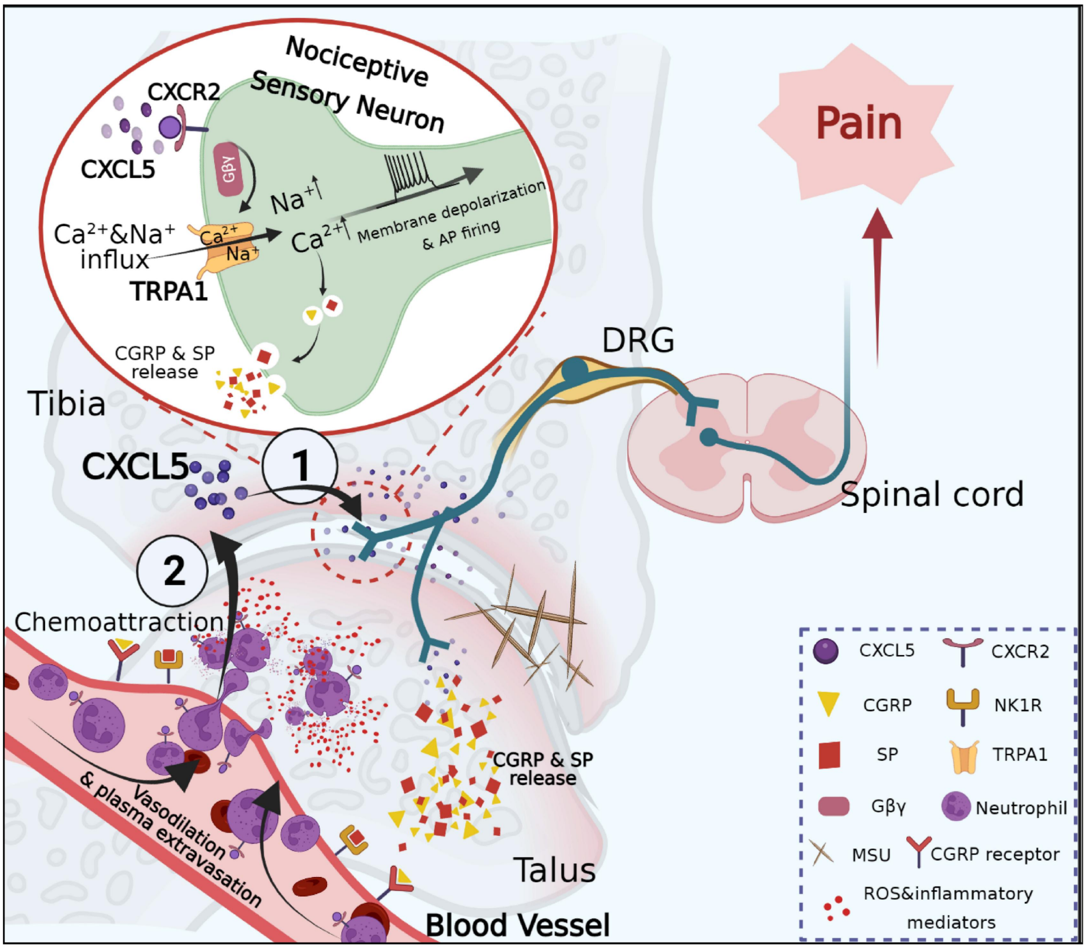

Suppl. Fig. 11 Proposed mechanisms for CXCL5-neuronal CXCR2-TRPA1 signaling to drive gout arthritis pain and joint inflammation. Step ①: CXCL5 released in the joint during gout arthritis acts on CXCR2 expressed in nociceptive sensory neurons to trigger TRPA1 activation via Gβγ signaling. TRPA1 activation results in membrane depolarization and action potential (AP) firing that produce pain signal. Multiple types of cells can produce CXCL5 during gout arthritis, including fibroblasts, macrophages and mast cells, etc. CXCR2's expression and coupling with TRPA1 are further increased in gout arthritis condition. CXCL5-induced TRPA1 activation in peptidergic nociceptors triggers Ca^{2+} influx that facilitates neuropeptide CGRP and SP release. Step ②: These neuropeptides act on CGRP receptor and SP NK1 receptor in nearby blood vessels to trigger potent vasodilation and plasma

extravasation that facilitate CXCL5-induced neutrophil chemotaxis from blood vessels into inflammatory site. The infiltrated neutrophils then release ROS and inflammatory mediators that further contribute to gout arthritis pain and joint inflammation. This figure was created with Biorender.com.

Tables S1. Reagents and products used in this study.

**Reagent or resource**	**Source**	**Identifier (Cat#)**
**Reagents**		
MSU crystal	Sigma	2875
Recombinant mCXCL1 Protein	Biolegend	573702
Recombinant mCXCL2 Protein	Biolegend	582502
Recombinant mCXCL5 Protein	Biolegend	573304
Recombinant hCXCL5 Protein	Biolegend	573406
Human Dorsal Root Ganglion Total RNA	Clontech	636150
Capsaicin	Abcam	ab141000
Allyl isothiocyanate	Sigma	W203408
SB225002	Glpbio	GC16465
Wortmannin	Glpbio	GC12338
U-73122	Glpbio	GC10451
U-0126	Glpbio	GC45099
H 89	Glpbio	GC19396
Ruthenium Red	Apexbio	B6740
iFluor® 594-WGA Conjugate	AAT Bioquest	25550
AMG9810	Tocris	2316
HC030031	Tocris	2896
Ionomycin	Beyotime	S1672
Lipofectamine 2000	Thermo Fisher	11668030
BIBN4096	MCE	HY-10095
L733060	Tocris	1145
Fura2-AM	Thermo Fisher	F1221
**Antibodies**		
Rabbit anti-CXCR2<br>(for IF&Co-IP)	Genetex	GTX14935
Rabbit anti-CXCR2<br>(for WB)	Huabio	ER1906-87
Rat anti-CXCL5	R&D	MAB433
Rat anti-CXCL1	R&D	MAB453
Rat anti-CXCL2	R&D	MAB452
Mouse anti-Gβ<br>(for WB&Co-IP)	Santa cruz	sc166123

Rabbit anti-Vimentin	Bioss	bs-0756R
Rabbit anti-Iba1	Wako	019-19741
Avidin Alexa Fluor™ 488 conjugate	Thermo Fisher	A21370
Rabbit anti-PGP9.5	Abcam	Ab108986
Mouse anti-CGRP	Sigma	C7113
Chicken anti-NF200	Abcam	Ab4680
IB4 FITC conjugated	Sigma	L2895
Rabbit Anti-GFP	Abcam	Ab6556
NeuroTrace 640/660 deep-red fluorescent Nissl stain	Invitrogen	N21483
Rabbit anti-TRPA1	Alomone	ACC-037
Mouse Anti-β-actin HRP conjugated	Huabio	M1210-5
PE Rat anti-Mouse Ly6G	BD	551461
BV421 Rat antiMouse CD182	BD	566622
APC Rat anti-Mouse CD11b	BD	553312
Anti-rabbit IgG, HRP-linked	CST	7074
Anti-mouse IgG, HRP-linked	CST	7076
Donkey Anti-Rabbit IgG H&L (Alexa Fluor® 488) preadsorbed	Abcam	Ab150065
Donkey Anti-Rabbit IgG H&L (Alexa Fluor® 647) preadsorbed	Abcam	Ab150067
Donkey Anti-Mouse IgG H&L (Alexa Fluor® 488) preadsorbed	Abcam	Ab150109
Goat Anti-Chicken IgY H&L (Alexa Fluor® 488) preadsorbed	Abcam	Ab150173
**Critical Commercial Assays**		
CXCL5 ELISA Kit	R&D Systems	MX000
CGRP ELISA Kit	Novus Biologicals	NBP3-00522
SP ELISA Kit	BBI Life Science	D751030
MPO activity Assay Kit	Elabscience	E-BC-K074-M
Immunoprecipitation Kit with Protein A+G Magnetic Beads	Beyotime	P2193M
Human Luminex® Discovery Assay	R&D Systems	LXSAHM
**Gene Symbol &Gene ID**	**Primer sequence (5' to 3')**	**Amplicon size (bp)**
β-actin 11461	F:5'-GTGCTATGTTGCTCTAGACTTCG-3' R:5'-ATGCCACAGGATTCCATACC-3'	174
Cxcl5 20311	F:5'-TGATCGCTAATTTGGAGGTGAT-3' R:5'-TAGCTTTCTTTTTGTCA	159

	CTGCC-3’	
Cxcr2<br>12765	F:5’-ATGCCCTCTATTCTGCC AGAT-3’<br>R:5’-GTGCTCCGGTTGTATA AGATGAC-3’	152
Cxcr1<br>227288	F:5’-TCTGGACTAATCCTGAG GGTG-3’<br>R:5’-GCCTGTTGGTTATTGGA ACTCTC-3’	111
Il-1β<br>16176	F:5’-CAACTGTTCCTGAACTC AACTG-3’<br>R:5’-GAAGGAAAAGAAGGT GCTCATG-3’	281
Tnf-α<br>21926	F:5’-ATGTCTCAGCCTCTTCT CATTC-3’<br>R:5’-GCTTGTCACTCGAATTT TGAGA-3’	179
Ccl2<br>20296	F:5’-TTAAAAACCTGGATCG GAACCAA-3’<br>R:5’-GCATTAGCTTCAGATTT ACGGGT-3’	121
Ccl3<br>20302	F:5’-TTCTCTGTACCATGACA CTCTGC-3’<br>R:5’-CGTGGAATCTTCCGGC TGTAG-3’	100
Cxcl1<br>14825	F:5’-AAGAATGGTCGCGAGG CTTG-3’<br>R:5’-AGGTGCCATCAGAGCA GTCT-3’	121
Cxcl2<br>114105	F:5’-GGTTGACTTCAAGAAC ATCCAG-3’<br>R:5’-TTGAGAGTGGCTATGA CTTCTG-3’	84
Il-6<br>16193	F:5’-CTCCCAACAGACCTGTC TATAC-3’<br>R:5’-CCATTGCACAACTCTTT TCTCA-3’	97
Il-10<br>16153	F:5’-TTCTTTCAAACAAAGG ACCAGC-3’<br>R:5’-GCAACCCAAGTAACCC TTAAAG-3’	81
Calca<br>12310	F:5’-AGCAGGAGGAAGAGCA GGA-3’<br>R:5’-CAGATTCCCACACCGC	71

	TTAG-3’	
Tac1<br>21333	F:5’-AGCCTCAGCAGTTCTTTGGA-3’<br>R:5’-TCTGGCCATGTCCATAAAGAG-3’	99
Ifn<br>15978	F:5’-GTGCTGCTGATGGGAGGAGATG-3’<br>R:5’-AGCCTGTTACTACCTGACACATTCG-3’	91
**Genotype Gene**	**Primer sequence (5’ to 3’)**	
Cxcr2 (Loxp) PCR1	F:5’-TGATGACTGAGGGAAATCTTGCAC-3’<br>R:5’-AGATAAAATGCAGGACTGTGAGGG-3’	
Cxcr2 (Loxp) PCR2	F:5’-GCATCGCATTGTCTGAGTAGGTG-3’<br>R:5’-CCCTCTTAGAGCAAGAGCCATAGC-3’	
Cxcr2 (KO) PCR1	F:5’-TGATGACTGAGGGAAATCTTGCAC-3’<br>R:5’-CCCTCTTAGAGCAAGAGCCATAGC-3’	
Cxcr2 (KO) PCR2	F:5’-ATTGCTGACCTGTTCTTTGCCC-3’<br>R:5’-ACCCGTAGCAGAACAGCATGATG-3’	
SNS Cre	F:5’-GAAAGCAGCCATGTCCAATTTACTGACCGTAC-3’<br>R:5’-GCGCGCCTGAAGATATAGAAGA-3’	
**Software and Algorithms**		
Flow Jo	Tree Star	Version 10
GraphPad Prism	GraphPad Software	Version 9
ANY-maze	Stoelting	Version 6.14

Table S2. Electrophysiological parameters of DRG neurons before and after CXCL5 application.

	Control	+CXCL5
Capacitance (pF)	19.6±0.8	-
RMP (mV)	-57.2±0.8	-52.4±0.9**
AP amplitude (mV)	109.8±1.8	105.3±1.9
AP frequency (Hz)	9.3±2.3	23.2±2.8**
AP half-peak width (ms)	2.1±0.1	2±0.1
AHP (mV)	15.0±1.4	17.5±1.6

Overshoot (mV)	51.9±1.3	47.9±1.4**
No. of neurons	17	-

RMP: resting membrane potential; AP: action potential; AHP: after hyperpolarization.
The data are shown as mean±SEM.
P values were determined by Student’s paired t test (two-tailed).
**$p < 0.01$ vs. control group.

Table S3. Characteristics of patients with acute gouty arthritis and healthy controls.

**Characteristics**	Healthy control (n=31)	Acute gouty arthritis (n=37)
Age (years)	32.19±1.23	33.86±1.6
Gender (male/female)	31/0	37/0
Serum uric acid (μmol/L)	331.97±8.17	566.31±6.08**

The data are shown as mean±SEM.
P values were determined by Student’s unpaired t test (two-tailed).
**$p<0.01$ *vs*. Healthy control group.
