# Supplementary material for: CXCL5 activates CXCR2 in nociceptive sensory neurons to drive joint pain and inflammation in experimental gouty arthritis
Source: Nat Commun. 2024 Apr 16;15:3263. doi: 10.1038/s41467-024-47640-7 (PMC11021482; doi:10.1038/s41467-024-47640-7)
Supplement: Supplementary file 3 — Reporting Summary [file 41467_2024_47640_MOESM3_ESM.pdf]

nature portfolio

Corresponding author(s): Boyi Liu

Last updated by author(s): Mar 1, 2024

Reporting Summary

Nature Portfolio wishes to improve the reproducibility of the work that we publish. This form provides structure for consistency and transparency in reporting. For further information on Nature Portfolio policies, see our Editorial Policies and the Editorial Policy Checklist.

Statistics

For all statistical analyses, confirm that the following items are present in the figure legend, table legend, main text, or Methods section.

n/a	Confirmed	
☐	☒	The exact sample size (*n*) for each experimental group/condition, given as a discrete number and unit of measurement
☐	☒	A statement on whether measurements were taken from distinct samples or whether the same sample was measured repeatedly
☐	☒	The statistical test(s) used AND whether they are one- or two-sided<br>*Only common tests should be described solely by name; describe more complex techniques in the Methods section.*
☐	☒	A description of all covariates tested
☐	☒	A description of any assumptions or corrections, such as tests of normality and adjustment for multiple comparisons
☐	☒	A full description of the statistical parameters including central tendency (e.g. means) or other basic estimates (e.g. regression coefficient) AND variation (e.g. standard deviation) or associated estimates of uncertainty (e.g. confidence intervals)
☐	☒	For null hypothesis testing, the test statistic (e.g. *F*, *t*, *r*) with confidence intervals, effect sizes, degrees of freedom and *P* value noted<br>*Give P values as exact values whenever suitable.*
☒	☐	For Bayesian analysis, information on the choice of priors and Markov chain Monte Carlo settings
☒	☐	For hierarchical and complex designs, identification of the appropriate level for tests and full reporting of outcomes
☐	☒	Estimates of effect sizes (e.g. Cohen's *d*, Pearson's *r*), indicating how they were calculated

Our web collection on statistics for biologists contains articles on many of the points above.

Software and code

Policy information about availability of computer code

Data collection	Zeiss ZEN 2.6 (fluorescent microscopy images), BGI-500 platform (RNA-seq), ANY-maze 6.13, DigiGait imaging system, Clampfit 10.2 (physiological recording), MetaFluor (Ca2+ imaging), HDOCK, open-source pymol, APBS.
Data analysis	Graphpad prism v9, Image J Fiji 2.9, R sudio 3.63, Flow Jo 10, AlphaView SA 3.5.0

For manuscripts utilizing custom algorithms or software that are central to the research but not yet described in published literature, software must be made available to editors and reviewers. We strongly encourage code deposition in a community repository (e.g. GitHub). See the Nature Portfolio guidelines for submitting code & software for further information.

Data

Policy information about availability of data

All manuscripts must include a data availability statement. This statement should provide the following information, where applicable:

- Accession codes, unique identifiers, or web links for publicly available datasets
- A description of any restrictions on data availability
- For clinical datasets or third party data, please ensure that the statement adheres to our policy

RNA-Seq data have been deposited in NCBI GEO under accession number GSE242872 (https://www.ncbi.nlm.nih.gov/geo/query/acc.cgi?acc=GSE242872).
A reporting summary for this article is available as a Supplementary Information file. The source data underlying all figures and supplementary figures are provided

in the Source Data file. The structures referred to in this study are available in the Protein Data Bank under the accession codes PDB 6V9W [http://doi.org/10.2210/pdb6V9W/pdb] and PDB 6RMV [http://doi.org/10.2210/pdb6RMV/pdb].

Research involving human participants, their data, or biological material

Policy information about studies with human participants or human data. See also policy information about sex, gender (identity/presentation), and sexual orientation and race, ethnicity and racism.

Reporting on sex and gender	Serum samples were obtained from leftover samples from 37 male patients and 31 male healthy controls.
Reporting on race, ethnicity, or other socially relevant groupings	*Please specify the socially constructed or socially relevant categorization variable(s) used in your manuscript and explain why they were used. Please note that such variables should not be used as proxies for other socially constructed/relevant variables (for example, race or ethnicity should not be used as a proxy for socioeconomic status).*<br>*Provide clear definitions of the relevant terms used, how they were provided (by the participants/respondents, the researchers, or third parties), and the method(s) used to classify people into the different categories (e.g. self-report, census or administrative data, social media data, etc.)*<br>*Please provide details about how you controlled for confounding variables in your analyses.*
Population characteristics	The patients with acute gout arthritis met the following criteria: male, aged 20-60 years old , admitted within 72 h after the onset of an acute gout attack, serum uric level ≥420 µM and without combined disease. Age-matched healthy males were included as healthy controls. Human DRG were collected from a male donor, with ages 67.
Recruitment	The serum samples were derived from leftover samples from diagnostic laboratory with sufficiently small harm risks to the participants.
Ethics oversight	The study was conducted following the Declaration of Helsinki and approved by the Ethics Committee of the Second Affiliated Hospital of Hebei Medical University (#2022- R282). The serum samples were derived from leftover samples from diagnostic laboratory with sufficiently small harm risks to the participants and the study is observational, then the Ethics Committee of the Second Affiliated Hospital of Hebei Medical University waived the requirement for informed consent. Human postmortem DRG samples was collected from body donations to the Dept. of Human Anatomy, Zhejiang Chinese Medical University, under the approval by Ethics Committee of Zhejiang Chinese Medical University (#ZJ-2161934-1).

Note that full information on the approval of the study protocol must also be provided in the manuscript.

Field-specific reporting

Please select the one below that is the best fit for your research. If you are not sure, read the appropriate sections before making your selection.

☒ Life sciences ☐ Behavioural & social sciences ☐ Ecological, evolutionary & environmental sciences

For a reference copy of the document with all sections, see nature.com/documents/nr-reporting-summary-flat.pdf

Life sciences study design

All studies must disclose on these points even when the disclosure is negative.

Sample size	Sample sizes were chosen based on similar previous studies utilizing these outcome measures (PMID: 23185004, 33827672, 28380690, 28992367,29563338 ,31351964)
Data exclusions	No data exclusions.
Replication	All attempts at replication were successful. Independent replicate numbers varied by experiment but in general were performed at least 3 times.
Randomization	Allocation to experimental groups was done randomly.
Blinding	The investigators were blinded to group allocation during data collection and analysis.

Reporting for specific materials, systems and methods

We require information from authors about some types of materials, experimental systems and methods used in many studies. Here, indicate whether each material, system or method listed is relevant to your study. If you are not sure if a list item applies to your research, read the appropriate section before selecting a response.

Materials & experimental systems

n/a	Involved in the study
☐	☒ Antibodies
☐	☒ Eukaryotic cell lines
☒	☐ Palaeontology and archaeology
☐	☒ Animals and other organisms
☒	☐ Clinical data
☒	☐ Dual use research of concern
☒	☐ Plants

Methods

n/a	Involved in the study
☒	☐ ChIP-seq
☐	☒ Flow cytometry
☒	☐ MRI-based neuroimaging

Antibodies

Antibodies used

For immunofluorescence, the following antibodies were used:
1st Ab:
Rabbit anti-CXCR2 (Genetex, GTX14935, 1:100),
Rabbit anti-PGP9.5(Abcam, Ab108986, 1:200),
Mouse anti-CGRP (Sigma, c7113, 1:2000),
IB4 FITC-conjugated (Sigma, L2895, 1:500),
Chicken anti-NF200 (Abcam, ab4680, 1:1000),
Nissl conjugated-640/660 deep-red fluorescent (Invitrogen, N21483, 1:500),
Rat anti-Ly6G (Thermo Fisher, 14-5931-82),
Rabbit anti-Vimentin (Bioss, bs-0756R, 1:500),
Rabbit anti-Iba1 (Wako, 019-19741, 1:500),
Avidin conjugated-Alexa Fluor™ 488 (Thermo Fisher, A21370, 1:500),
Rat anti-CXCL5 (R&D, MAB433, 1:200),
Mouse anti-GFAP (CST, 3670, 1:500),
Mouse anti-NeuN (Abcam, Ab104224, 1:500),
Rabbit anti-GFP (Abcam, Ab6556, 1:1000).
2nd Ab:
Donkey Anti-Rabbit IgG H&L Alexa Fluor® 488 (Abcam, Ab150065, 1:1000),
Donkey Anti-Rabbit IgG H&L Alexa fluor® 647 (Abcam, Ab150067, 1:1000),
Donkey Anti-Mouse IgG H&L Alexa Fluor® 488 (Abcam, Ab150109, 1:1000),
Donkey Anti-chicken IgY H&L Alexa Fluor" 488(Abcam, Ab150173, 1:1000),
Goat Anti-Rat IgG H&L Alexa Fluor® 488 (Abcam, ab150157, 1:1000),
Donkey Anti-Mouse IgG H&L Alexa Fluor® 594 (Abcam, Ab150108, 1:1000).

For WB and Co-IP, the following antibodies were used:
1st Ab: Rabbit ant-CXCR2 (Genetex, GTX 14935, 1:500),
Rabbit anti-CXCR2 (Huabio, ER1906-87, 1:1000),
Rabbit anti-TRPA1 (Alomone, ACC-037, 1:200),
Mouse anti-Gβ (Santa cruz, sc166123, 1:500),
Mouse anti-β-actin HRP conjugated (Huabio, M1210-5, 1:5000).
2nd Ab:
Anti-rabbit IgG HRP-linked (CST, 7074, 1:5000)
Anti-mouse IgG HRP-linked (CST, 7076, 1:5000).

For FACS, the following antibodies were used:
PE Rat anti-Mouse Ly6G (BD, 551461, 1:100),
BV421 Rat anti-Mouse CD182 (BD, 566622, 1:50),
APC Rat anti-Mouse CD11b (BD, 553312, 1:100).

Validation

We performed testing as follows: Negative control: absence of staining in tissue not expressing from Cxcr2-/- mice. Positive control: replication of staining in tissue known to express the CXCR2 and previously published (PMID: 31125710). Other antibodies used in this study were obtained from commercial suppliers and were validated by the manufacturers for their specific application. Please see the corresponding manufacturer datasheets or product details section on the webpages for reference, verified reactivity species and validation.
Antibody for IF:
anti-PGP9.5: On the product website, it is indicated that this antibody has 69 citations of papers that have used it. In the present study, we did not take additional steps to validate this antibody.
anti-CGRP: : On the product website, this antibody has been used in 2 published papers. We did not take additional steps to validate this antibody.
IB4 FITC-conjugated: On webstite (‘bing.com/academic/’), this antibody has been used in least 5 published papers (PMID: 25538155, 23872594, 23749568 and 26242746). We did not take additional steps to validate this antibody.
Nissl conjugated-640/660 deep-red fluorescent: We used this antibody in our previous study (PMID: 27821781). Additionally, on the product website, it is indicated that this antibody has 20 citations of papers that have used it, In the present study. we did not take additional steps to validate this antibody.
anti-Ly6G: On the product website, it is indicated that this antibody has 360 citations of papers that have used it. In the present study, we did not take additional steps to validate this antibody.
anti-Vimentin: On the product website, it is indicated that this antibody has 106 citations of papers that have used it. In the present study, we did not take additional steps to validate this antibody.

anti-Iba1: We used this antibody in our previous study (PMID: 38363535). Additionally, on the product website, it is indicated that this antibody has 4160 citations of papers that have used it. In the present study, we did not take additional steps to validate this antibody.
Avidin conjugated-Alexa Fluor 488: On the product website, it is indicated that this antibody has 19 citations of papers that have used it. In the present study, we did not take additional steps to validate this antibody.
anti-GFAP: We used this antibody in our previous study (PMID: 37158939). Additionally, on the product website, it is indicated that this antibody has 764 citations of papers that have used it. In the present study, we did not take additional steps to validate this antibody.
anti-NeuN: We used this antibody in our previous study (PMID: 37158939, PMID: 31105572). Additionally, on the product website, it is indicated that this antibody has 483 citations of papers that have used it. In the present study, we did not take additional steps to validate this antibody.
anti-GFP: On the product website, it is indicated that this antibody has 1204 citations of papers that have used it. In the present study, we did not take additional steps to validate this antibody.

Antibody for WB&Co-IP:
anti-TRPA1: We used this antibody in our previous study (PMID: 30990108, 30990108). Additionally, on the product website, itisindicated that this antibody has 87 citations of papers that have used it, In the present study. we did not take additional steps ovalidate this antibody.
anti-Gβ: on the product website, it is indicated that this antibody has 9 citations of papers that have used it in the present study, we did not take additional steps to validate this antibody.

Antibody for FACS
PE Rat anti-Mouse Ly6G: on the product website, it is indicated that this antibody has 1 citations of papers that have used it in the present study, we did not take additional steps to validate this antibody.
BV421 Rat anti-Mouse CD182: on the product website, it is indicated that this antibody has 3 citations of papers that have used it in the present study, we did not take additional steps to validate this antibody.
APC Rat anti-Mouse CD11b: on the product website, it is indicated that this antibody has 9 citations of papers that have used it in the present study, we did not take additional steps to validate this antibody.

Eukaryotic cell lines

Policy information about cell lines and Sex and Gender in Research

Cell line source(s)	HEK293T cells were purchased from ATCC (#CRL-3216).
Authentication	HEK293T cells were purchased from ATCC. No further authentication procedures were performed.<br>HEK293T: https://www.atcc.org/products/crl-3216.
Mycoplasma contamination	Cells were not mycoplasma positive.
Commonly misidentified lines<br>(See ICLAC register)	No commonly misidentified cell lines were used in this study.

Animals and other research organisms

Policy information about studies involving animals; ARRIVE guidelines recommended for reporting animal research, and Sex and Gender in Research

Laboratory animals	Species:Mice; Strains: Cxcr2-/- and Cxcr2fl/fl mice (GemPharmatech, #T037599, #T051899), Ly6g-IRES-GFP mice (Shanghai Model Organisms Center,NM-KI-220554) Trpv1-/- and Trpa1-/- mice were provided by Prof. Zhen-zhong Xu (Zhejiang University School of Medicine). SNS-Cre mice were provided by Professor Xu Zhang (Chinese Academy of Sciences, Shanghai, China). All mice used on a C57BL/6 background. Age:6-8 weeks. The mice were maintained in a pathogen-free environment on a 12-hr light/dark cycle at controlled temperature (24±2°C) and humidity (50-60%).
Wild animals	No wild animals were used in this study.
Reporting on sex	MSU-induced joint pain and inflammation experiment: both male and female mice.<br>Other experiments: male mice.<br>Detailed information can be found in the methods section.
Field-collected samples	This study does not involve samples collected from the field.
Ethics oversight	All animal experiments were approved by the Laboratory Animal Management and Welfare Ethical Review Committee of Zhejiang Chinese Medical University (Permission No.: #IACUC-20190819-04).

Note that full information on the approval of the study protocol must also be provided in the manuscript.

Flow Cytometry

Plots

Confirm that:

☒ The axis labels state the marker and fluorochrome used (e.g. CD4-FITC).

☒ The axis scales are clearly visible. Include numbers along axes only for bottom left plot of group (a 'group' is an analysis of identical markers).

☒ All plots are contour plots with outliers or pseudocolor plots.

☒ A numerical value for number of cells or percentage (with statistics) is provided.

Methodology

Sample preparation	The ankle joints were collected, diced and digested with 2 mg/ml dispase and 1 mg/ml collagenase type 1 in RPMI 1640+10% fetal bovine serum (FBS) for 1 h at 37°C. The cells were filtered through a cell strainer with a 70 µm nylon mesh and washed with RPMI 1640+10% FBS. Cells were then stained with a standard panel of immunophenotyping antibodies. Detailed information can be found in the methods section.
Instrument	FACS Canto II Cytometry (BD Biosciences, USA)
Software	FlowJo software (BD Biosciences, USA)
Cell population abundance	Under the used threshold settings the proportion of intact protoplasts was roughly 30 % of a recorded events. The amount of fluorescent-positive cell population varied from 15 % to 40 % depending on the group analysed (see Fig. 9).
Gating strategy	We first identify cells of interest based on size and granularity (complexity) through FSC vs SSC. Subsequently, cell populations were gated by negative control samples to determine the fluorescence intensity threshold for positive expression.

☒ Tick this box to confirm that a figure exemplifying the gating strategy is provided in the Supplementary Information.
